# Examining the Relationship Between Community Resources and Early Disability Identification: Variation by Child Race or Ethnicity

**DOI:** 10.1007/s11121-025-01829-4

**Published:** 2025-08-16

**Authors:** J. LoCasale-Crouch, T. Lenahan, Z. Shea, J. Whittaker, Y. Zhang, B. Duyile, Y. Xu, A. Williford

**Affiliations:** 1 School of Education, Virginia Commonwealth University, Richmond, VA USA; 2 Graduate School of Education, Harvard University, Cambridge, MA USA; 3 School of Education and Human Development, University of Virginia, Charlottesville, VA USA

**Keywords:** Early childhood, Community resources, Disability identification, Equitable access

## Abstract

Early identification and intervention support for children with disabilities improve their cognitive, educational, and social outcomes. Studies show that disability identification varies by child race and ethnicity, with children from historically marginalized populations being less likely to be identified during early childhood, where identification tends to happen in the community. One major factor that varies across communities is their resources, broadly defined as the environmental, social, and economic factors within a geographically defined area. While extensive evidence exists noting inequitable distribution of community resources by race and ethnicity, little research has examined whether community resources are associated with differential early disability identification rates. This study explored the association between the availability of community resources and early disability identification and whether it varied by child race or ethnicity. Leveraging 2019 statewide data collected through the Virginia Department of Education (VDOE), we combined information about 91,210 incoming kindergarteners with the Child Opportunity Index 2.0 (COI), a measure of community resources known to be associated with child development. After controlling for multiple covariates, children entering kindergarten at schools in higher-resourced communities were more likely to be identified with a disability prior to kindergarten. Although access to community resources and early disability identification rates varied by child race and/or ethnicity, the interaction effect was not significant. This study has implications for viewing community resources as potential malleable factors to address children’s varying needs prior to the start of formal schooling.

Children’s development is shaped by the systems around them, including the communities in which they reside. Early community support via individualized intervention and treatment for children with disabilities can improve their cognitive, educational, and social outcomes (Guralnick, 2017; Ruane & Carr, 2019). Thus, communities serve as a critical prevention and intervention factor for enhancing the lives of children with disabilities. Accessing early support is contingent on early identification in the community, and prior research shows that disability identification rates vary by race. Although, in later schooling, minoritized students tend to be over-represented in disability identification (National Center for Learning Disabilities, 2020), studies of identification for early intervention find that children from historically marginalized populations are less likely to be identified (Gallegos et al., 2021; Guarino et al., 2010; Morgan et al., 2015). This early under-identification limits access to supportive services that can potentially change children’s developmental trajectories. Thus, further exploration of the intersection between early disability identification and race is needed.

Given that communities are an early and critical system children experience, examining their role in early disability identification is warranted. One major factor that varies across communities is their resources, broadly defined as the environmental, social, and economic factors within a geographically defined area. Previous research shows that community resources play a significant role in explaining health disparities (Braveman et al., 2011), and lack of access to community resources has been found to be a key contributor to race or ethnicity achievement gaps. For example, a recent study showed an association between community resources and children’s school readiness at the start of kindergarten, with minoritized children living in areas with lower community resources, and these lower resource areas were associated with school readiness (Lenahan et al., 2022). Further, in more segregated neighborhoods, community resources were even more strongly associated with children’s school readiness. Given that minoritized children are more likely to have fewer access to community resources (Acevedo-Garcia et al., 2020; Hardy et al., 2021), it is possible that community resources may contribute to the differential early disability identification rates by child race or ethnicity. This study, then, aims to examine the association between the availability of community resources and early disability identification and whether this association varies by children’s race or ethnicity.

## The Importance of Early Disability Identification and Variation by Child Race or Ethnicity

Accurate and early disability identification and appropriate intervention services are vital to children’s development. Research indicates that high-quality early intervention services have meaningful short-term effects on the developmental outcomes of children with identified disabilities and their families (Rose et al., 2014; Ruane & Carr, 2019). Additionally, early disability identification is related to timely intervention which may contribute to long-term effects, particularly for children from families with limited resources (Chen et al., 2022). Across diverse groups and different service levels, early intervention has been connected to increased cognitive ability such as academic achievement and IQ (Guralnick, 2017; Kim et al., 2018; Waters et al., 2020), social-emotional development (Green et al., 2015, 2017), and middle to long-term effects on decreasing the number of students placed in special education (Carlson et al., 2009; Shinde & Maeda, 2019).

The timely start of early intervention is contingent on identification. For example, an early and accurate diagnosis of autism spectrum disorder (ASD) enables families to learn about their child’s developmental challenges, cope with caregiving demands, seek appropriate services, and obtain genetic counseling (Shattuck et al., 2009). Additionally, in a 20-year follow-up research study of children with developmental delays from families with limited resources, findings suggested that intervention prior to starting formal schooling mattered for their long-term outcomes. Specifically, they found that children who received intervention services through preschool, compared with similar families who did not receive intervention services, had higher academic achievement, lower rates of grade retention, and decreased referral for special education services (Reynolds et al., 2001). And Chen and colleagues, 2022) found that the timing of early intervention varied by family income and race or ethnicity; however, less is known about how race or ethnicity intersects with community resources and early disability identification.

The positive outcomes associated with early intervention support are dependent on identification. How identification occurs varies by age. In early childhood, identification typically occurs following a community referral (i.e., child care provider and physician) to the locally designated organization, which can then initiate the process for a child to be assessed. Once children start school, the identification process is often initiated by the school. Disability identification rates, then, tend to increase over time. The national disability identification rate before school starts is difficult to calculate due to variations in locality reporting; however, federal data includes required reporting once children begin services within the public system. In the 2022–2023 school year, the rate of children aged 3–21 receiving special education and/or related services for a disability was 15% of public school students, with rates highest among American Indian/Alaska Native (19%), Black (17%) and lowest for Asian (8%) students (National Center for Education Statistics, 2024). While multiple studies found an overrepresentation of minoritized students in later special education processes in the school setting (Aron & Loprest, 2012; NCLD, 2020; Sullivan, 2011), other studies have found that children from historically marginalized populations are less likely than White children to be identified early when it comes from the community (Constantino et al., 2020; Gallegos et al., 2021; Morgan et al., 2015). Thus, an examination of factors that might explain differences in early identification rates by child race and ethnicity is needed.

## The Role of Community Resources in Fostering Children’s Development and Variation by Child Race or Ethnicity

Ecological system theory highlights communities as one of the key systems that impact human development (Bronfenbrenner, 1994). Indeed, extensive research shows that community conditions (e.g., air quality and green space) are critical predictors of both adult and children’s health and development (Ayscue et al., 2017; Braveman et al., 2011; Jilani et al., 2021; Lenahan et al., 2022; Martinez-Sande et al., 2022; NSCDC, 2023). Even after controlling for individual characteristics, community factors such as poverty level continue to be consistently linked with child development (Ayscue et al., 2017; Brooks-Gunn et al., 2021; Martínez–Sande et al., 2022). Communities, then, are an important context to consider when examining supports available to foster children’s development.

Recent research suggests that defining community resources using only socioeconomic factors does not capture important variations in living conditions (Wei et al., 2021), nor the potentially malleable policy levers that might influence the resources a community has available to foster children’s development. Thus, Acevedo-Garcia et al. (2020) created the Child Opportunity Index (COI), containing 29 indicators measuring place-based resources, to better reflect community environmental, social, and economic factors associated with children’s development. In addition to research associating the COI with life expectancy and intergenerational socioeconomic mobility, recent research has begun to examine the COI in association with aspects of children’s development (Acevedo-Garcia et al., 2020). For example, in a recent state-wide study of children’s school readiness, Lenahan and colleagues (2022) found that, after controlling for individual characteristics, a child in a higher-resourced community as measured by the COI was more likely to be school ready than a similar child in a lower-resourced community. The COI, then, appears to be capturing unique community investments that relate to immediate child development as well as longer-term health and well-being.

Further, recent research suggests that associations between community resources and children’s development vary considerably by race and ethnicity, with minoritized populations being over-represented in low-resourced areas and under-represented in high-resourced ones (Acevedo-Garcia et al., 2020; Hardy et al., 2021). Further, in the Lenahan et al.’s (2022) study noted above, not only did access to community resources vary by race and ethnicity, children within a more segregated area were more sensitive to changes in community resources than those in less segregated areas. Specifically, children in segregated Black/Hispanic communities saw the greatest gains to their school readiness likelihood from an increase in COI. This suggests that paying attention to both resources and race within a community is warranted.

### The Current Study

Despite extensive research suggesting links between community context and young children’s development, surprisingly little research has examined its association with early disability identification, which directly relates to the timing of receiving developmentally enhancing support. Given the opportunities to enhance children’s development provided through provision of intervention when a disability or delay is identified early, understanding factors related to early identification are critical to explore. Past research points to communities playing a critical role in children’s development, noting that community resources vary by race or ethnicity. The purpose of this study, then, is to examine the association between community resources and disability identification at the beginning of kindergarten and whether it varies by the child’s race or ethnicity. Specifically, this study aims to answer the following research questions:

1. What is the association between availability of community resources and children’s disability identification at the beginning of kindergarten?
2. Does the strength of the association between availability of community resources and disability identification at the beginning of kindergarten vary by child race or ethnicity?

This research will contribute to the existing literature by expanding our understanding of whether the availability of community resources is associated with early disability identification, and if so, whether the strength of this association varies based on a child’s race or ethnicity. Findings will inform our understanding of variation in early identification by community context and race, providing potential malleable pathways to target in future intervention and policy work.

## Method

Data used in this study were part of the statewide kindergarten readiness assessment in Virginia initiative overseen by the Virginia Department of Education (VDOE). Study approval was obtained through the university IRB and data agreements with the VDOE. A series of steps were taken to extract child-level kindergarten data, including demographics, and whether they were identified with a disability at the start of kindergarten in the fall of 2019. Data were available for nearly all children entering kindergarten. For this analysis, the Fall 2019 data were used for several reasons. First, these data included over 99% of the expected kindergarten population and their disability identification status at this time. Second, the population reflects the Commonwealth’s racial (20.4% Black), ethnic (17.2% Hispanic), and socioeconomic (37.6% economically disadvantaged) diversity. Third, this data predated the COVID-19 pandemic.

### Participants

In Fall 2019, 91,210 kindergartners across 1106 schools participated in the statewide kindergarten readiness assessment. Following the model of prior work (Lenahan et al., 2022), we used school attendance boundaries as the geographic organizer for communities (details to follow). Attendance boundary information was missing for 68 schools, and four schools were dropped as less than half of their expected child population was represented in the dataset. In addition, we excluded children without disability information (*n* = 998). Race or ethnicity included Asian, Black or African American, Hispanic/Latino of any race, White, and two or more races, and Other (American Indian or Alaska Native, Native Hawaiian, or other Pacific Islander). While there was sufficient power to detect overall effects, some cell sizes became very small when examining interactions by race (discussed further in the Analytic Approach section). Thus, the following racial groups were excluded from the analysis: Asian, two or more races, and Other (*n* = 11,932 observations dropped). The final analytic sample included 72,872 children, about 80% of the original sample. See Table 1 for information. Compared to the original sample, this sample has a higher percentage of Black, Hispanic/Latino, and White children and those identified as an English language learner, from an economically disadvantaged family, and with a disability.

**Table 1:** Disability identification rates by child demographic groups

Child demographics	*n*	%	Disability-identified *n*	Disability-identified, %
Total children	72,872		6894	9.5
Total schools	1034			
**Gender**				
Female	35,363	48.5	2038	5.8
Male	37,509	51.5	4856	12.9
**Race or ethnicity**				
Black	17,697	24.3	1632	9.2
Hispanic	15,019	20.6	1205	8.0
White	40,156	55.1	4057	10.1
**Economically disadvantaged**				
Disadvantaged	28,667	39.3	3373	11.8
Not disadvantaged	44,205	60.7	3521	8.0
**EL status**				
EL	10,195	14.0	934	9.2
Not EL	62,677	86.0	5960	9.5
**Pre-K experience**				
No PK	16,961	23.3	454	2.7
Headstart	3533	4.8	254	7.2
Public	25,826	35.4	5636	21.8
Private/daycare	24,778	34.0	514	2.1
Dept. of defense	551	0.8	18	3.3
Family home	1223	1.7	18	1.5

^*^998 and 12,315 children dropped due to missing characteristic data and low observation counts, respectively

In our analysis sample, about 48.5% of the children were female, 55.1% were White, 24.3% were Black, and 20.6% were Hispanic. More than one third of children (39.3%) were economically disadvantaged, and 14% of the sample were English language learners. About 9.5% of the sample were identified with a disability at kindergarten entrance.

### Measures

#### Disability Status

A child was considered to have a disability if they had an individualized education plan (IEP) for a federally identified disability at the start of kindergarten. Identified disabilities included autism, developmental delay, emotional disturbance, hearing impairments, intellectual disabilities, multiple disabilities, orthopedic impairments, other health impairments, specific learning disabilities, speech or language impairments, traumatic brain injury, and visual impairments. Based on small samples within certain disabilities that would limit our ability to examine individual categories, we created a single disability variable, assigning a value of 1 if a child had an IEP. Because these children already had an IEP in place at the beginning of kindergarten, this means they would have engaged in the identification process prior to the start of kindergarten.

#### Community Resources

The *Child Opportunity Index* (COI) 2.0 was used to measure community resources (Acevedo-Garcia et al., 2020). The COI 2.0 is a census tract-level index of 29 indicators that reflect place-based resources such as access and quality of early childhood education, green space, access to healthy foods, toxin-free environments, and socioeconomic resources. Data in the COI come from the American Community Survey as well as open-source and government datasets. The individual indicators were weighted according to correlations between the indicators and several health outcomes. Z-scores were then created and aggregated into one overall state-normed composite score, with higher scores indicating higher resourced communities (diversitydatakids.org, 2023). Prior research shows COI related to a host of critical child outcomes, including health (NSCDC, 2023) and school readiness (Lenahan et al., 2022).

Following Lenahan et al. (2022), this study used school boundaries as the geographical organizer for the communities where children lived. School Attendance Boundaries (SAB) provide a way to delineate communities around an elementary school. SABs, or school feeder zones, are the geographical areas served by a school. The National Center for Education Statistics’ (NCES, 2018) School Attendance Boundary Survey is one of the most complete and up-to-date sources of SABs available. To join the COI at the census-tract level with the SABs, the geographical map of the 2015 SAB Survey was overlaid with the 2015 census tract map using geospatial analysis and calculated the percent of each tract contained within an SAB (NCES, 2018; U.S. Census Bureau, 2016). These percentages were then used to weight the overall COI score of each census tract contained within an SAB, to calculate an SAB-wide weighted average COI score.

#### Child Characteristics

Child demographic characteristics included race or ethnicity (i.e., Asian, Black or African American, Hispanic/Latino of any race, White, two or more races, and Other (American Indian or Alaska Native, Native Hawaiian, or other Pacific Islander). Preschool experience was a state-assigned code to identify a child’s most recent pre-k experience. Children from low-income backgrounds were identified as economically disadvantaged if at any point during the school year the child was eligible for Free/Reduced Meals or Medicaid and/or received TANF. English learner (EL) children were identified using the VDOE EL Code.

#### Analytic Approach

For the first research question, we examined whether the availability of community resources was associated with a child’s likelihood of being identified with a disability at the beginning of kindergarten. To answer this, a series of logistic regressions were estimated. The key explanatory variable was the standardized weighted average SAB COI score.

The base model specification, given in the following equation generated the results for research question 1. This model demonstrated the likelihood child *i* in SAB *s* was identified with a disability as a function of the weighted average COI in the child’s SAB as well as other characteristics of the child. The coefficient ${\beta }_{1}$ was the coefficient of interest. It represented the change in the likelihood of being identified with a disability associated with an additional one standard deviation increase to COI, controlling for other child characteristics (i.e., race or ethnicity, gender, pre-K experience, English learner status, and economically disadvantaged status).

$$PR(DI_{is})=\frac1{1+e^{\beta X}}\;\mathrm{where}\;BX=\beta_0+\beta_1COI_s+\theta^\prime(child\,Chars)_i$$

The second research question focused on whether the strength of the association varied by a child’s racial/ethnic identity. For this analysis, an interaction term was added to the base model between the included race or ethnicity of child, *i*, and COI to assess whether the strength of the association between COI and disability identification at the beginning of kindergarten varied by child race or ethnicity.

One assumption of the logit model is linearity between the log odds of the dependent variable and continuous variables. Each model only included one continuous variable, SAB-overall COI score. Nonlinearity was tested by running models including both the score’s natural and higher order forms. Neither of these tests supported the presence of functional form misspecification. The presence of outliers and multicollinearity was also tested using the Pregibon Delta Beta Statistic test and Variance Inflation Factor, respectively. Both tests strongly suggested that neither was a concern (Akinwande et al., 2015).

Moreover, a critical consideration throughout the analyses was maintaining adequate statistical power to detect effects. Despite a nearly complete sample of the population of kindergarten children in Virginia in fall 2019, the limited number of children identified with a disability imposed meaningful constraints on our analyses. Keeping this in mind, numerous power analyses were conducted on both the sample and the coefficients of the model.

Van Smeden et al. (2019) identified the number of predictors, the total sample size, and the proportion of events within the binary outcome as factors relevant to optimizing the minimum detectable effect size for logistic regression estimates. Power calculations testing for required sample sizes were performed in STATA using estimated effect sizes, conventional beta (80%) and alpha (0.05) parameters, and the proportion identified with a disability. The results of these calculations found that this study had sufficient statistical power to detect the estimated main effects on our variable of interest (COI). However, when testing estimates for interaction terms between COI and child race, the analytic sample only exceeded the necessary observations for Black, Hispanic/Latino, and White children. Therefore, underpowered groups were excluded.

#### Reporting Results

To facilitate model interpretation, results are reported as odds-ratios, which show the change in the likelihood of the child being identified with a disability by kindergarten relative to a baseline, holding other child characteristics constant. In all models, standard errors are reported clustered at the SAB level. Furthermore, we present predicted probabilities of the average child being identified with a disability at the beginning of kindergarten when in a SAB with a COI one-half of a standard deviation below the mean. In other words, the change in the likelihood of identification for an average child from a one standard deviation increase to COI. These predicted probabilities were provided for each racial/ethnic identity.

We use two definitions to construct the change in probability for the “average” child of different racial/ethnic groups, each providing different insights. The Grand mean fixes all covariates at the analytic sample mean *across* racial/ethnic groups. This method allows for simple comparisons across identities as other covariates are equal (i.e., *ceteris peribus*). Conversely, the Group mean holds all covariates at their mean *within* a given child’s race or ethnicity. This constructs a more accurate representation of the average, say, Black child, as they may systematically differ from the average child within the full sample. While we present both methods, given our interest in cross-racial comparisons of the effects of community conditions, our preferred model uses Grand means.^1^

## Results

Our descriptive analysis revealed clear disparities in access to community resources among different racial groups. White children generally experienced higher-resourced communities compared to Black and Hispanic children. Specifically, as community resources increased from very low (1–20), low (> 20–38), medium (> 38–54), high (> 54–75), and to very high (> 75–100), the percentage of White children gradually increased from 36, 48, 54, and 59 to 75%. In contrast, the pattern for Black children was the opposite. The percentage of Black children decreased as resource levels increased, ranging from 49, 30, 21, and 16 down to 9%. Hispanic children displayed a more evenly distributed pattern across the levels, with 14% at very low, 21% at low, 24% at medium, 25% at high, and 17% at very high levels. Among the child population, 10.1% of White children, 9.2% of Black children, and 8% of Hispanic children were identified with a disability by kindergarten.

### What Is the Association Between Availability of Community Resources and a Child Being Identified with a Disability By Kindergarten?

Community resources as defined by the SAB COI were, on average, positively associated with a child’s likelihood of being identified with a disability by kindergarten. The analysis found that, holding all other covariates constant, a child in a higher-resourced SAB was nearly 34% more likely (*p* < 0.001) to be identified with a disability by kindergarten than a similar child in a lower-resourced SAB (see Table 2, Model 2). This change corresponds to an increase in expected disability identification from 4.2 to 5.6% (1.4 points or 33.3%).

**Table 2:** Estimated coefficients as odds ratios from models predicting disability status

	Model 1	Model 2
	COI-alone	Full
SAB COI (standardized)	0.946**	1.339***
	(0.017)	(0.029)
Black		0.586***
		(0.026)
Hispanic		0.594***
		(0.029)
Female		0.408***
		(0.012)
Econ disadvantaged		0.952
		(0.035)
English learner		0.849**
		(0.047)
No PK		0.093***
		(0.006)
Headstart PK		0.288***
		(0.024)
Private PK		0.046***
		(0.003)
DoD PK		0.089***
		(0.022)
Family day home		0.041***
		(0.010)
Constant	0.105***	0.594***
	(0.002)	(0.022)
Observations	72,872	72,872
Pseudo *R*^2^	0.000409	0.201

This table reports estimates from regressing whether a child was identified as having a disability on the neighborhood conditions surrounding their school, along with other covariates. Covariates include race or ethnicity, sex, economically disadvantaged, and prior pre-K experience. Standard errors clustered at the SAB level are in parentheses. ***, **, *, and + mark estimates statistically different from zero at the 99.9, 99, 95, and 90 percent confidence levels, respectively

### Does the Strength of the Relationship Between Availability of Community Resources and a Child Being Identified with a Disability by Kindergarten Vary by Their Race or Ethnicity?

The second research question asks whether the above relationship varies by race or ethnicity. Table 3 (Model 2) shows that, controlling for other child characteristics, Black and Hispanic children were 41.4 and 40.6% less likely (*p* < 0.001) to be identified as having a disability by kindergarten than a similar White child. Then, we ran a model interacting SAB COI with a child’s racial/ethnic identity to test for disparate effects of neighborhood resources across racial groups. Results in Table 4 display the associated change in a child’s likelihood of being identified as having a disability by kindergarten for Black and Hispanic children relative to a similar White child at different COI values—first at the mean weighted average COI (main effects) and then again in a more resourced SAB (interaction effect). The main effect coefficients for children are almost identical to those reported in Table 3 (Model 2) showing White children as significantly more likely to be identified than Black or Hispanic children. The interaction results show that, while all children are more likely to be identified in higher-resourced communities, we found no statistically significant difference in the change across racial groups.

**Table 3:** Change in predicted disability status likelihood by interaction term

		Predicted disability status probability		Change in predicted probability from + 1 SD COI	
		At COI = − 0.5	At COI = + 0.5	Percentage points	%
Overall					
	All children	4.2	5.6	1.4***	33.3
Grand^a^					
	Race or ethnicity				
	Black	3.2	4.4	1.2***	37.5
	Hispanic	3.3	4.2	0.9***	27.3
	White	5.4	7.0	1.6***	29.6
Group^b^					
	Race or ethnicity				
	Black	5.2	7.2	2.0***	38.5
	Hispanic	3.6	4.6	1.0***	27.8
	White	4.2	5.5	1.3***	31.0

***p < 0.001; **p < 0.01; *p < 0.05; + *p* < 0.1

^a^All other variables in the models held constant at the mean among all children

^b^All other variables in the models held constant at the mean among children in SABs of the specific segregation type and level

**Table 4:** Selected estimated coefficients as odds ratio from models predicting disability status

SAB COI (standardized)	1.324***
	(0.037)
Black^✝^	0.601***
	(0.026)
Hispanic^✝^	0.596***
	(0.029)
Black * SAB COI (standardized)	1.062
	(0.050)
Hispanic * SAB COI (standardized)	0.980
	(0.044)
Observations	72,872
Pseudo R2	0.201

All variables include child characteristics as listed in Model 2 of Table 2

Robust form in parentheses

***p < 0.001; **p < 0.01; *p < 0.05; + *p* < 0.1

^✝^Coefficient represents effect size at mean COI (main effects)

## Discussion

The purpose of this study was to examine the association between availability of community resources and disability identification at the beginning of kindergarten and whether it varied by child’s race or ethnicity. Several key findings emerged. First, children in lower-resourced communities were less likely to be identified with a disability by kindergarten, limiting their potential access to early intervention support. Second, although race or ethnicity differences emerged in both early identification rates and access to community resources, the strength of the association between community resources and rate of identification by kindergarten did not vary by child race or ethnicity. This study has implications for examining the community in which a child resides as a key input to their development. Points will be further elaborated on below.

### Early Disability Identification and Access to Community Resources Varies by Race and Ethnicity

Reflecting patterns in prior work examining variation in early disability identification rates by race (Chen et al., 2022; Guarino et al., 2010; Morgan et al., 2015), we found that Black and Hispanic children were identified with a disability prior to kindergarten at a lower rate than their White peers. This raises the question about equity in the disability identification process and highlights the importance of considering the role of intersectionality in understanding the disparities that exist in the early disability identification process. This finding suggests a closer examination of assessment practices, cultural competence among evaluators, and the availability of appropriate screening tools for diverse populations. It is critical that we bring awareness to the disparities, as the inability to receive an appropriate diagnosis can impact a child’s ability to receive early interventions (Morgan et al., 2015). The disparity in access to early individualized support can exacerbate educational inequalities and perpetuate cycles of underachievement among minoritized children (Cooc, 2022).

Similarly, access to community resources varied by race and ethnicity. This mirrors others’ research that consistently finds Black and Hispanic children are over-represented in lower-resourced communities and White children over-represented in higher-resourced ones (Acevedo-Garcia et al., 2020; Hardy et al., 2021; Lenahan et al., 2022). And these racial differences in access to resourced communities persist, even when controlling for family income (Hardy et al., 2021). These disparities in access to community resources point to underlying structural inequalities within communities. Research continues to highlight the crucial role of community resource access in shaping health outcomes and overall well-being. Communities with more access to resources such as quality healthcare, education, employment opportunities, and safe housing tend to experience better health outcomes and disparities in access contribute to poorer outcomes such as educational attainment, economic stability, and life expectancy (Baciu et al., 2017). Thus, implementation of policies that address these structural barriers and create more equitable opportunities for minority communities is needed.

While access to resourced communities varied by race the strength of the relationship between those resources and early disability identification did not. This means that there may be other family, service, intersecting, and contextual factors outside of the presence of resources in a community that can impact a child’s ability to be identified with a disability by kindergarten (Sapiets et. al., 2021). Thus, examining the multifaceted dynamics that lead to disability identification is warranted to ensure equitable access to early intervention and support services for all children, regardless of their racial or socioeconomic background. Future studies should examine the interactive relationship between race and ethnicity, disability, and community resources to further understand their relationship in order to address them.

### More Resourced Communities Show Higher Rates of Early Disability Identification

The other key study finding points to a positive association between community resources and children’s early disability identification rates. Interestingly, studies in other countries have found a higher prevalence of disabilities in areas with fewer resources (Fortune et al., 2020). Thus, what appears consistent is that community resources matter but may function differently depending on the timing (i.e., early identification) and cultural context. Given that early identification is key to timely intervention (Chen et al., 2022), this finding suggests that considering enhanced screening for communities with fewer resources may be beneficial. Not only do lower-resourced communities need support to identify children with disabilities, they also need to have the quality support services to enhance development. As noted by the World Health Organization (2012), identification needs to be accompanied by services and effective follow-up to ensure they are being utilized with the intended impact. Including this ongoing support will ensure that identified children receive the support they need.

What is less clear is why and how community resources play such an important role in disability identification and potential access to developmentally enhancing supports prior to kindergarten. One consideration may be due to how variable the referral process for identification prior to kindergarten is between communities, especially in those with lower resources. Prior to kindergarten, pre-referral is highly related to family resources, including access to and engagement with early care programs or health professionals, to engage the referral procedure. While the early intervention (EI, birth to 3) referral process usually starts with a health or child care provider, families in lower-resourced communities may have very limited access to these agencies. As a result, a child’s disability may not be identified until later when they enter school. Once children are potentially school eligible and exhibiting potential delays, they may become eligible for early childhood special education (ECSE, 3–5) through a referral through Child Find. Families in higher-resourced communities, however, tend to have more access to EI/ECSE resources than families in lower-resourced communities (Xu & Filler, 2005). Thus, while ECSE services are offered at no cost for families, schools in lower-resourced communities may struggle with the comprehensive evaluation procedure following the referral due to the shortage of high-quality interdisciplinary personnel.

Thus, considering how to address community resource gaps is needed to better enhance developmental supports available for young children. For example, strengthening professional training and resources for educators, healthcare providers, and other professionals in lower-resourced communities is a critical step. Research suggests that limited provider training and knowledge about early disability indicators contribute to disparity in identification (Lynch et al., 2024), emphasizing the need for professional training in addition to addressing the community resource gaps. Additionally, having a robust care coordination system in child care and schools can help bridge gaps in early identification by ensuring that children’s health and social needs are met through integrated, systematic support networks (Francis et al., 2021). At the same time, care needs to be taken to ensure disability identification does not exacerbate discrimination and exclusion. Not only do systems need support for equitable identification processes for all children, but also policies need to be enacted to ensure accessible, affordable, responsive, high-quality care is available regardless of the communities in which one lives (WHO, 2012).

### Limitations and Future Directions

Although findings advance our understanding of the associations between community resources and disability identification by kindergarten, the study includes a few notable limitations. First, the percentage of children identified for special education, even in a large sample, was under 10%, and thus the small sample may limit the full ability to detect effects. Further, excluding sub-groups of children from the analyses, even though the small sample cells required it, limits the generalizability of findings to those who are Black, Hispanic, or White. Additionally, the aggregation of community resources to the school level may have resulted in the data not fully reflecting the child’s community experience. Meaning, some children might have been in a neighborhood with higher, or lower, community resources than the average of the school attendance boundary reflected, potentially losing meaningful variation. Finally, having access to or availability of community resources does not guarantee their use. Thus, future work would benefit from examining availability and use of community resources together.

Despite these limitations, this study provides insights into how communities serve as an important component for prevention and intervention to enhance the lives and well-being of children with disabilities. Thus, policy makers and educators can consider ways in which access to developmentally supportive resources across systems can occur. Future work should also consider additional aspects of communities or particular types of disabilities that intersect with race or ethnicity and how children’s health and development occur over time. With the support and understanding from this work, we can aim to provide all children and families, particularly from under-resourced communities, the timely and appropriate services needed to thrive.

## Data Availability

VDOE data are available by request to them.

## Footnotes

1 The results from each prediction differ because they correspond to two different points along the nonlinear estimates produced by the logit model. Each set of predictions has unique advantages and disadvantages. The Grand means allow for comparisons where the only difference is a child’s race, but it does not account for the different average profile of a child within each racial/ethnic category. On the other hand, using Group means limits the ability to compare effects across racial categories but allows for testing the predicted change for the average student within that racial group.

## Declarations

### Disclaimer

The opinions expressed are those of the authors and do not represent the views of the Virginia Department of Education.

## References

[CR1] Acevedo-Garcia, D., Noelke, C., McArdle, N., Sofer, N., Hardy, E. F., Weiner, M., Baek, M., Huntington, N., Huber, R., & Reece, J. (2020). Racial and ethnic inequities in children’s neighborhoods: Evidence from the new Child Opportunity Index 2.0. *Health Affairs,**39*(10), 1693–1701. 10.1377/hlthaff.2020.00735 33017244 10.1377/hlthaff.2020.00735

[CR2] Akinwande, M. O., Dikko, H. G., & Samson, A. (2015). Variance inflation factor: As a condition for the inclusion of suppressor variable (s) in regression analysis. *Open Journal of Statistics,* (07), 754. 10.4236/ojs.2015.57075

[CR3] Aron, L., & Loprest, P. (2012). Disability and the education system. *The Future of Children*, 97–122. https://www.jstor.org/stable/41475648 10.1353/foc.2012.0007 22550687

[CR4] Ayscue, J., Frankenberg, E., & Siegel-Hawley, G. (2017). The complementary benefits of racial and socioeconomic diversity in schools. Research Brief No. 10. *National Coalition on School Diversity. *https://www.diverseschools.org/research-briefs

[CR5] Baciu, A., Negussie, Y., Geller, A., & Weinstein, J. N. (2017). The root causes of health inequity. In National Academies of Sciences, Engineering, and Medicine & Committee on Community-Based Solutions to Promote Health Equity in the United States (Eds.), *Communities in action: Pathways to health equity* (pp. 99–184). National Academies Press.10.17226/24624 28418632

[CR6] Braveman, P., Egerter, S., & Williams, D. R. (2011). The social determinants of health: Coming of age. *Annual Review of Public Health,**32*(1), 381–398. 10.1146/annurev-publhealth-031210-101218 21091195 10.1146/annurev-publhealth-031210-101218

[CR7] Bronfenbrenner, U. (1994). Ecological models of human development. In T. Husen & T. Postlethwaite (Eds.),* International encyclopedia of education *(2nd ed., Vol. 3, pp. 1643–1647)*.* Pergamon Press.

[CR8] Brooks-Gunn, J., Klebanov, P., Liaw, F. R., & Duncan, G. (2021). Toward an understanding of the effects of poverty upon children. In *Children of poverty* (pp. 3–41). Routledge.

[CR9] Carlson, E., Daley, T., Bitterman, A., Heinzen, H., Keller, B., Markowitz, J., & Riley, J. (2009). Early school transitions and the social behavior of children with disabilities: Selected findings from the Pre-Elementary Education Longitudinal Study (PEELS). NCSER Publication No. 2009-3016. *National Center for Special Education Research*. https://ies.ed.gov/ncser/2025/01/20093016-pdf

[CR10] Chen, C. C., Cheng, S. L., Xu, Y., Spence, C., Zhang, F., & Adams, N. B. (2022). Child developmental and special education service receipt: The intersection of health and poverty. *Disability and Health Journal,**15*(3), Article 101269. 35131214 10.1016/j.dhjo.2022.101269

[CR11] Constantino, J. N., Abbacchi, A. M., Saulnier, C., Klaiman, C., Mandell, D. S., Zhang, Y., … & Geschwind, D. H. (2020). Timing of the diagnosis of autism in African American children. *Pediatrics, 146*(3). 10.1542/peds.2019-3629 10.1542/peds.2019-3629 PMC7461218 32839243

[CR12] Cooc, N. (2022). Disparities in general education inclusion for students of color with disabilities: Understanding when and why. *Journal of School Psychology,**90*, 43–59. 10.1016/j.jsp.2021.10.002 34969487 10.1016/j.jsp.2021.10.002

[CR13] diversitydatakids.org. (2023). *Child Opportunity Index 2.0 database*. Retrieved from https://data.diversitydatakids.org/dataset/coi20-child-opportunity-index-2-0-database. Accessed 23 Jul 2021

[CR14] Fortune, N., Singh, A., Badland, H., Stancliffe, R. J., & Llewellyn, G. (2020). Area-level associations between built environment characteristics and disability prevalence in Australia: An ecological analysis. *International Journal of Environmental Research and Public Health,**17*(21), 7844. 33114716 10.3390/ijerph17217844 PMC7662552

[CR15] Francis, L., DePriest, K., Sharps, P., Wilson, P., Ling, C., Bowie, J., & Thorpe, R. J., Jr. (2021). A mixed-methods systematic review identifying, describing, and examining the effects of school-based care coordination programs in the US on all reported outcomes. *Preventive Medicine,**153*, Article 106850. 10.1016/j.ypmed.2021.106850 34662597 10.1016/j.ypmed.2021.106850

[CR16] Gallegos, A., Dudovitz, R., Biely, C., Chung, P. J., Coker, T. R., Barnert, E., Guerrero, A. D., Szilagyi, P. G., & Nelson, B. B. (2021). Racial disparities in developmental delay diagnosis and services received in early childhood. *Academic Pediatrics,**21*(7), 1230–1238. 10.1016/j.acap.2021.05.008 34020100 10.1016/j.acap.2021.05.008 PMC9169674

[CR17] Green, J., Charman, T., Pickles, A., Wan, M. W., Elsabbagh, M., Slonims, V., Taylor, Carol, McNally, Janet, Booth, Rhonda, Gliga, Teodora, Jones, Emily J H., Harrop, Clare, Bedford, Rachael, & Johnson, M. H. (2015). Parent-mediated intervention versus no intervention for infants at high risk of autism: A parallel, single-blind, randomized trial. *The Lancet Psychiatry,**2*(2), 133–140. 10.1016/S2215-0366(14)00091-1 26359749 10.1016/S2215-0366(14)00091-1 PMC4722333

[CR18] Green, J., Pickles, A., Pasco, G., Bedford, R., Wan, M. W., Elsabbagh, M., ... & McNally, J. (2017). Randomized trial of a parent‐mediated intervention for infants at high risk for autism: Longitudinal outcomes to age 3 years. *Journal of Child Psychology and Psychiatry*, *58*(12), 1330–1340. 10.1111/jcpp.12728 10.1111/jcpp.12728 PMC5724485 28393350

[CR19] Guarino, C. M., Buddin, R., Pham, C., & Cho, M. (2010). Demographic factors associated with the early identification of children with special needs. *Topics in Early Childhood Special Education,**30*(3), 162–175. 10.1177/0271121409349273

[CR20] Guralnick, M. J. (2017). Early intervention for children with intellectual disabilities: An update. *Journal of Applied Research in Intellectual Disabilities,**30*(2), 211–229. 26764896 10.1111/jar.12233

[CR21] Hardy, R., Boch, S., Keedy, H., & Chisolm, D. (2021). Social determinants of health needs and pediatric health care use. *The Journal of Pediatrics,**238*, 275–281. 10.1016/j.jpeds.2021.07.056 34329688 10.1016/j.jpeds.2021.07.056

[CR22] Jilani, M. H., Javed, Z., Yahya, T., Valero-Elizondo, J., Khan, S. U., Kash, B., ... & Nasir, K. (2021). Social determinants of health and cardiovascular disease: Current state and future directions towards healthcare equity. *Current Atherosclerosis Reports, 23*, 1–11. 10.1007/s11883-021-00949-w 10.1007/s11883-021-00949-w 34308497

[CR23] Kim, S. H., Bal, V. H., & Lord, C. (2018). Longitudinal follow-up of academic achievement in children with autism from age 2 to 18. *Journal of Child Psychology and Psychiatry,**59*(3), 258–267. 10.1111/jcpp.12808 28949003 10.1111/jcpp.12808 PMC5819744

[CR24] Lenahan, T., LoCasale-Crouch, J., Chamberlain, C., Williford, A., Downer, J., Whittaker, J., & Miller, L. (2022). Examining the association between neighborhood conditions and school readiness across low and highly segregated school attendance boundaries. *Frontiers in Education,**7*, Article 932558. 10.3389/feduc.2022.932558

[CR25] Lynch, P., Nabwera, H. M., Babikako, H. M., Rasheed, M., Donald, K. A., Mbale, E. W., Stockdale, Elizabeth, Chand, Prem, Van den Heuvel, Meta, Kakooza Mwesige, Angelina, & Gladstone, M. (2024). Experiences of identifying pre-school children with disabilities in resource limited settings – an account from Malawi, Pakistan and Uganda. *Disability & Society,**39*(8), 2053–2073. 39045395 10.1080/09687599.2023.2181769 PMC11265222

[CR26] Martínez-Sande, P. A., Pacheco, K. C., Martínez-González, M. B., & Chajin, L. H. (2022). Cognitive development of children in vulnerable contexts: The role of psychosocial intervention. *Early Child Development and Care,**192*(11), 1744–1751. 10.1080/03004430.2021.1932863

[CR27] Morgan, P. L., Farkas, G., Hillemeier, M. M., Mattison, R., Maczuga, S., Li, H., & Cook, M. (2015). Minorities are disproportionately underrepresented in special education: Longitudinal evidence across five disability conditions. *Educational Researcher,**44*(5), 278–292. 10.3102/0013189X15591157 27445414 10.3102/0013189X15591157 PMC4950880

[CR28] National Center for Education Statistics. (2018). *The School Attendance Boundary Survey (SABS)*. SABS/Home. https://nces.ed.gov/programs/edge/sabs. Accessed 20 Dec 2020

[CR29] National Center for Education Statistics. (2024). Students with disabilities. *Condition of Education*. U.S. Department of Education, Institute of Education Sciences. Retrieved [March 15th, 2025], from https://nces.ed.gov/programs/coe/indicator/cgg.

[CR30] National Center for Learning Disabilities. (2020). Significant disproportionality in special education: Current trends and actions for impact. https://ncld.org/wp-content/uploads/2023/07/2020-NCLD-Disproportionality_Trends-and-Actions-for-Impact_FINAL-1.pdf

[CR31] National Scientific Council on the Developing Child. (2023). Place matters: The environment we create shapes the foundations of healthy development. Working Paper No. 16. Center on the Developing Child at Harvard University. https://developingchild.harvard.edu/resources/working-paper/place-matters-the-environment-we-create-shapes-the-foundations-ofhealthy-development/

[CR32] Reynolds, A. J., Temple, J. A., Robertson, D. L., & Mann, E. A. (2001). Long-term effects of an early childhood intervention on education achievement and juvenile arrest. *JAMA,**285*(18), 2339–2346. 10.1001/jama.285.18.2339 11343481 10.1001/jama.285.18.2339

[CR33] Rose, L., Herzig, L. D., & Hussey-Gardner, B. (2014). Early intervention and the role of pediatricians. *Pediatrics in Review,**35*(1), e1–e10. 10.1542/pir.35-1-e1 24385566 10.1542/pir.35-1-e1

[CR34] Ruane, A., & Carr, A. (2019). Systematic review and meta-analysis of Stepping Stones Triple P for parents of children with disabilities. *Family Process,**58*(1), 232–246. 10.1111/famp.12352 29520764 10.1111/famp.12352

[CR35] Sapiets, S. J., Totsika, V., & Hastings, R. P. (2021). Factors influencing access to early intervention for families of children with developmental disabilities: A narrative review. *Journal of Applied Research in Intellectual Disabilities,**34*(3), 695–711. 10.1111/jar.12852 33354863 10.1111/jar.12852 PMC8246771

[CR36] Shattuck, P. T., Durkin, M., Maenner, M., Newschaffer, C., Mandell, D. S., Wiggins, L., Lee, Li-Ching., Rice, Catherine, Giarelli, Ellen, Kirby, Russell, Baio, Jon, Pinto-Martin, Jennifer, & Cuniff, C. (2009). Timing of identification among children with an autism spectrum disorder: Findings from a population-based surveillance study. *Journal of the American Academy of Child and Adolescent Psychiatry,**48*(5), 474–483. 10.1097/CHI.0b013e31819b3848 19318992 10.1097/CHI.0b013e31819b3848 PMC3188985

[CR37] Shinde, S. K., & Maeda, Y. (2019). National trends in changes in special education classification among pre-elementary education children. *Exceptionality*, *27*(1), 32–46. https://doi-org.proxy.library.vcu.edu/10.1080/09362835.2017.1355802

[CR38] Sullivan, A. L. (2011). Disproportionality in special education identification and placement of English language learners. *Exceptional Children,**77*(3), 317–334. 10.1177/001440291107700304

[CR39] U.S. Census Bureau. (2016). Tiger/Line Shapefiles: 2015 census tracts [Data set]. https://www.census.gov/geographies/mappingfiles/time-series/geo/tiger-line-file.html

[CR40] Van Smeden, M., Moons, K. G., de Groot, J. A., Collins, G. S., Altman, D. G., Eijkemans, M. J., & Reitsma, J. B. (2019). Sample size for binary logistic prediction models: Beyond events per variable criteria. *Statistical Methods in Medical Research,**28*(8), 2455–2474. 10.1177/0962280218784726 29966490 10.1177/0962280218784726 PMC6710621

[CR41] Waters, C. F., Amerine Dickens, M., Thurston, S. W., Lu, X., & Smith, T. (2020). Sustainability of early intensive behavioral intervention for children with autism spectrum disorder in a community setting. *Behavior Modification,**44*(1), 3–26. 30009626 10.1177/0145445518786463

[CR42] Wei, W. S., McCoy, D. C., Busby, A. K., Hanno, E. C., & Sabol, T. J. (2021). Beyond neighborhood socioeconomic status: Exploring the role of neighborhood resources for preschool classroom quality and early childhood development. *American Journal of Community Psychology,**67*(3–4), 470–485. 10.1002/ajcp.12507 33780018 10.1002/ajcp.12507

[CR43] World Health Organization. (2012). Early childhood development and disability: A discussion paper. https://iris.who.int/bitstream/handle/10665/75355/9789241504065_eng.pdf?sequence=1

[CR44] Xu, Y., & Filler, J. (2005). Linking assessment and intervention for developmental/functional outcomes of premature, low-birth-weight children. *Early Childhood Education Journal,**32*(6), 383–389. 10.1007/s10643-005-0008-4

